# Two alternative pathways for generating transmissible prion disease *de novo*

**DOI:** 10.1186/s40478-015-0248-5

**Published:** 2015-11-10

**Authors:** Natallia Makarava, Regina Savtchenko, Ilia V. Baskakov

**Affiliations:** Center for Biomedical Engineering and Technology, University of Maryland School of Medicine, 111 S. Penn St., Baltimore, MD 21201 USA; Department of Anatomy and Neurobiology, University of Maryland School of Medicine, Baltimore, MD USA

## Abstract

**Introduction:**

Previous studies established that prion disease with unique strain-specific phenotypes could be induced by *in vitro*-formed recombinant PrP (rPrP) fibrils with structures different from that of authentic prions, or PrP^Sc^. To explain the etiology of prion diseases, new mechanism proposed that in animals the transition from rPrP fibrils to PrP^Sc^ consists of two main steps: the first involves fibril-induced formation of atypical PrPres, a self-replicating but clinically silent state, and the second consists of atypical PrPres-dependent formation of PrP^Sc^ via rare deformed templating events.

**Results:**

In the current study, atypical PrPres with characteristics similar to those of brain-derived atypical PrPres was generated *in vitro*. Upon inoculation into animals, *in vitro*-generated atypical PrPres gave rise to PrP^Sc^ and prion disease with a phenotype similar to those induced by rPrP fibrils. Significant differences in the sialylation pattern between atypical PrPres and PrP^Sc^ suggested that only a small sub-fraction of the PrP^C^ that is acceptable as a substrate for PrP^Sc^ could be also recruited by atypical PrPres. This can explain why atypical PrPres replicates slower than PrP^Sc^ and why PrP^Sc^ outcompetes atypical PrPres.

**Conclusions:**

This study illustrates that transmissible prion diseases with very similar disease phenotypes could be produced via two alternative procedures: direct inoculation of recombinant PrP amyloid fibrils or *in vitro*-produced atypical PrPres. Moreover, this work showed that preparations of atypical PrPres free of PrP^Sc^ can give rise to transmissible diseases in wild type animals and that atypical PrPres generated *in vitro* is an adequate model for brain-derived atypical PrPres.

**Electronic supplementary material:**

The online version of this article (doi:10.1186/s40478-015-0248-5) contains supplementary material, which is available to authorized users.

## Introduction

Prions are proteinaceous infectious agents that are underlying causes of fatal neurodegenerative diseases known as prion diseases or transmissible spongiform encephalopathies (TSE) [1]. Prions consist of misfolded, aggregated states of the normal, cellular form of the prion protein (PrP^C^). Prion diseases can arise spontaneously, be inherited or be acquired through transmission [1]. Prions spread between organisms or from cell to cell by replicating their disease-specific misfolded structures via a template-assisted mechanism [2]. This mechanism postulates that PrP^Sc^ template recruits and converts PrP^C^ expressed by a host into PrP^Sc^. According to this mechanism, the folding pattern of a newly formed PrP^Sc^ accurately replicates that of the PrP^Sc^ template, and prion replication exhibits high fidelity [2].

Recent studies demonstrated that in the presence of certain cellular components such as RNA and/or lipids, highly pure recombinant PrP converts into authentic PrP^Sc^ conformations *in vitro* that can effectively induce transmissible prion diseases in animals [3–6]. At the same time, transmissible prion disease could also be induced by recombinant PrP (rPrP) amyloid fibrils formed *in vitro* in the absence of any co-factors although at low efficiency [7–12]. Bearing in mind that the structures of rPrP fibrils produced *in vitro* were found to be fundamentally different from that of PrP^Sc^ [13, 14], these studies raised the possibility that an unexplored mechanism responsible for the etiology of prion diseases exists [15, 16]. According to this mechanism, referred to as deformed templating, a self-replicating state with one folding pattern can seed structurally different self-replicating states with alternative folding patterns when exposed to a new replication environment [16]. The newly generated self-replicating states that fit well to the new environment outcompete the original template. A new replication environment might include, but is not limited to, new biochemical or cellular conditions, a new substrate with altered amino acid sequence or posttranslational modifications, or presence of prion inhibitors [17–22]. In direct support of the deformed templating mechanism, molecular imaging revealed that switching between alternative folding patterns can occur within individual PrP amyloid fibrils when amyloid seeds were exposed to heterologous substrate *in vitro* [23]. In another illustration of the deformed templating mechanism, novel, structurally altered self-replicating PrP^Sc^ states that were absent in the original seeding material emerged upon changes to the biochemical environment, which involved a depletion of RNA from protein cyclic amplification reactions and subsequent addition of RNA to the reactions [17]. In addition, upon change in pH, non-infectious fibrils of recombinant fungal prion protein HET-s with a stacked β-sheet architecture gave rise to the alternative, infectious state of HET fibrils with a β-solenoid structure [24].

**Fig. 1:**
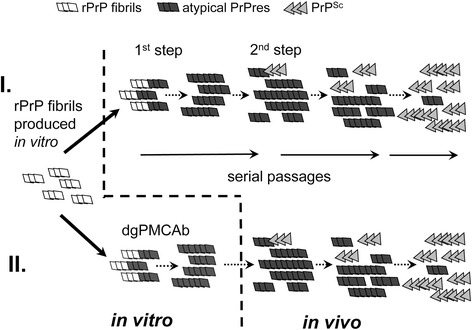
Schematic illustration of two alternative pathways for generating PrP^Sc^. The first pathway was established in previous studies [11, 12], it involves direct inoculation of *in vitro*-generated rPrP fibrils into animals and consists of two main steps. In a first step, rPrP fibrils seeded atypical PrPres, a transmissible form of PrP that replicates silently without causing clinical disease. In a second step, atypical PrPres produces PrP^Sc^ in rare and stochastic seeding events that are described by a deformed templating mechanism [16]. PrP^Sc^ replicates faster than atypical PrPres and eventually replaces it during serial passages. An alternative pathway is under investigation by the current work. *In vitro*-generated rPrP fibrils were used to seed serial dgPMCAb that produced fibril-induced atypical PrPres, which was inoculated into animals

Previous studies proposed that evolution of authentic PrP^Sc^ from rPrP fibrils consists of two main steps [11, 12] (Fig. 1). The first step involves formation of atypical PrPres, a self-replicating state that is triggered in animals upon inoculation of rPrP fibrils. While atypical PrPres is transmissible, it is clinically silent and not neurotoxic [25]. In a second step, atypical PrPres give rise to PrP^Sc^ in rare deformed templating events that are believed to be stochastic in nature. Because atypical PrPres and PrP^Sc^ are significantly different in structure [11, 12, 26, 27], a transition from the former to the latter is believed to represent the major barrier to the evolution of authentic PrP^Sc^.

The current work is inspired by recent findings that atypical PrPres with characteristics similar to those of brain-derived atypical PrPres can be generated *in vitro* by seeding Protein Misfolding Cyclic Amplification reactions with beads (PMCAb) using rPrP fibrils (Fig. 1). Three questions have been asked in the current work. The first is whether atypical PrPres generated *in vitro* gives a rise to PrP^Sc^ and prion disease in animals (Fig. 1). The second is whether the strain-specific disease phenotype associated with *in vitro*-generated atypical PrPres is similar to those of synthetic strains produced by direct inoculation of animals by rPrP fibrils. The third is whether evolution of authentic PrP^Sc^ and development of prion diseases can be accelerated if the first of the two main steps, i.e. formation of atypical PrPres, is accomplished *in vitro*.

## Materials and methods

### Expression and purification of rPrP and formation of rPrP fibrils

Syrian hamster full-length rPrP encompassing residues 23–231 was expressed and purified according to a previously described procedure [28] with minor modifications [10]. Lyophilized rPrP was dissolved in 5 mM HEPES, pH 7.0, immediately before use. To form fibrils, a mixture of 0.5 mg/ml rPrP with 50 mM MES, pH 6.0, and 2.0 M or 0.5 M guanidine hydrochloride (GdnHCl) was incubated at 37 °C under continuous agitation. Amyloid formation was confirmed by thioflavin T fluorescence assay as described previously [28]. Fibrils were dialyzed against 10 mM sodium acetate, pH 5.0 for storage. If required, fibrils were heat-treated in PBS, pH 7.4 in the presence of 5 mg/ml BSA (Sigma, Cat. #A3294) or 5 % brain homogenate prepared from healthy hamsters (see below) as described before [10, 29].

### Preparation of 10 % brain homogenate (BH)

10 % (wt/vol) brain homogenates were prepared in PBS, pH 7.4, using glass/Teflon homogenizers attached to a cordless 12 V compact drill (Ryobi) as previously described [30].

### Protein misfolding cyclic amplification with beads (PMCAb)

10 % normal brain homogenate (NBH) from healthy hamsters was prepared as described previously [12] and used as a substrate for PMCAb [31]. For the first round, 10 μl of water-diluted rPrP fibrils were added to 90 μl of NBH resulting in the 5 μg/ml final rPrP concentration in the reaction. For the reactions seeded with brain-derived PrP^Sc^, 10 μl of scrapie BH from inoculated animals were diluted in PBS to achieve the desired final concentration and added to 90 μl of NBH. The standard sonication program consisted of 20 s sonication pulses at ~150 W applied every 20 min during a 24 h period. For each subsequent round, 10 μl of the reaction from the previous round were added to 90 μl of fresh substrate. Each PMCAb reaction was carried out in the presence of two 3/32” Teflon beads (amazonsupply.com). To analyze production of PK-resistant PrP material in PMCAb, 10 μl of sample were supplemented with 5 μl of SDS and 5 μl of PK to a final concentration of SDS and PK of 0.25 % and 50 μg/ml, respectively, followed by incubation at 37 °C for 1 h. The digestion was terminated by addition of SDS-sample buffer and heating the samples for 10 min in a boiling water bath.

### Protein misfolding cyclic amplification with partially deglycosylated substrate (dgPMCAb) and generation of atypical PrPres *in vitro*

**Fig. 2:**
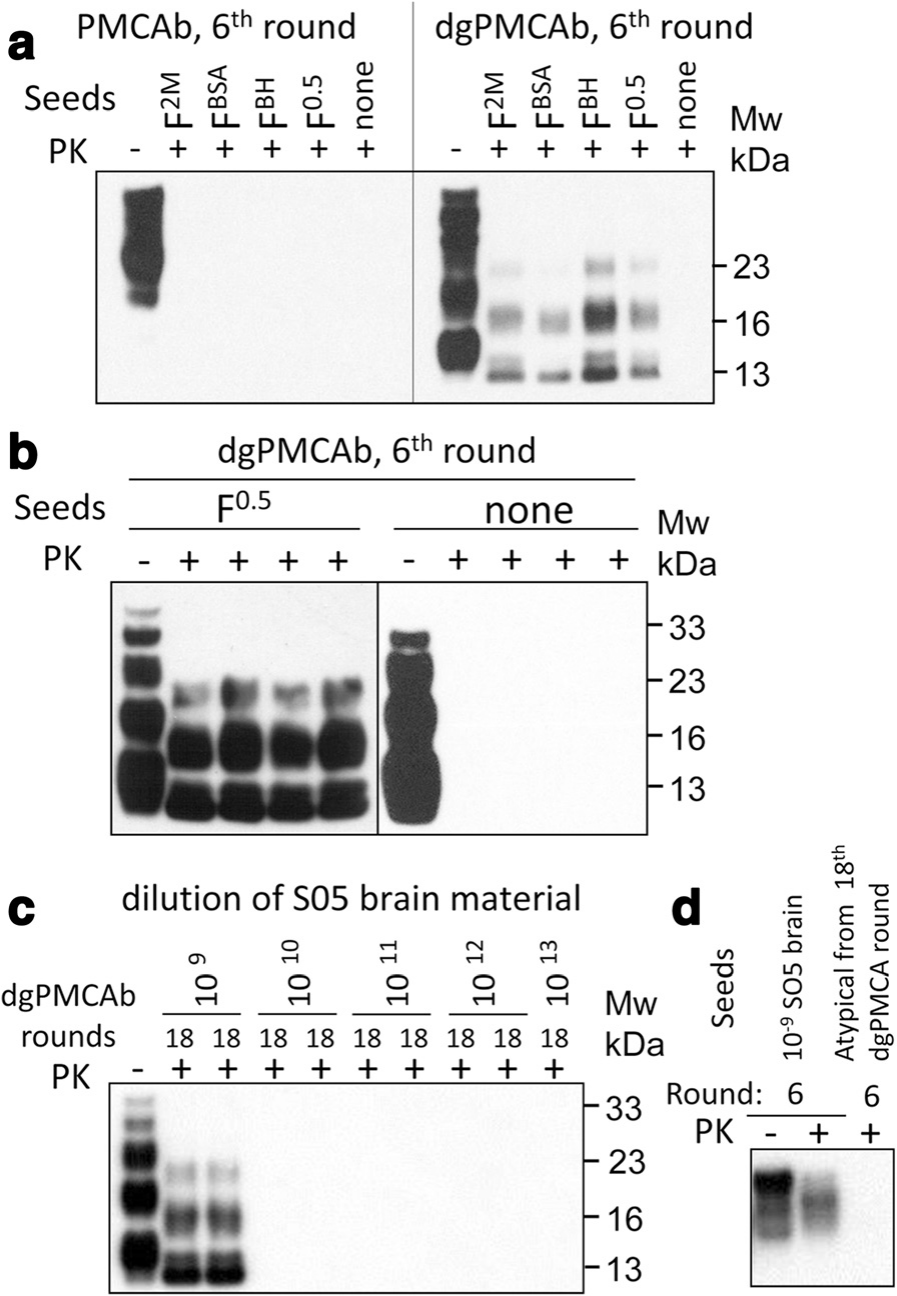
Generating rPrP fibril-induced atypical PrPres in dgPMCAb. **a** Analysis of PK-resistant materials produced in 6^th^ round of serial PMCAb or dgPMCAb reactions seeded with four preparations of rPrP fibrils or non-seeded reactions by Western blot. F^2M^ or F^0.5^ are fibrils generated in 2 M or 0.5 M GdnHCl, respectively, as previously described [11]. F^BSA^ or F^NBH^ are fibrils annealed in BSA or NBH as previously described [10, 29]. **b** Analysis of PK-resistant materials produced in 6^th^ round of serial dgPMCAb reactions seeded with four independent preparations of F^0.5^ fibrils or non-seeded reactions by Western blot. **c** Establishing a limiting dilution for atypical PrPres in S05 brain material. S05 brain material was subjected to 10-fold serial dilutions for up to 10^13^-fold and used for seeding serial dgPMCAb, using a procedure previously described [34]. Western blot analysis of 18^th^ serial dgPMCA rounds demonstrates that 10^9^-fold diluted S05 brain material was the last dilution that contained atypical PrPres material. **d** To confirm that preparation of brain-derived atypical PrPres subjected to 18^th^ dgPMCA rounds lacks PrP^Sc^, serial PMCAb reactions were seeded with the products of 18^th^ dgPMCAb round or 10^9^-fold diluted S05 brain material, then subjected to six serial PMCAb rounds and analyzed by Western blot. PK-resistant bands at 23, 16 and 13 kDa represent di-, mono- and unglycosylated atypical PrPres, respectively. Western blots in panels **a**-**c** were stained with SAF-84 antibody and in panel **d** with 3F4 antibody

To produce substrate for dgPMCAb, 10 % NBH from healthy hamsters prepared for PMCAb was treated with peptide-*N*-glycosidase F (PNGase F) (New England BioLabs, glycerol-free) as follows. After preclearance of NBH at 500 × *g* for 2 min, 1500 U/ml PNGase F was added to the supernatant, and the reaction was incubated on a rotator at 37 °C for 5 h. The resulting substrate was used in dgPMCAb with sonication conditions described for PMCAb. To produce brain-derived atypical PrPres, dgPMCA substrate was treated with RNase A (Sigma, catalog no. R4875) for 1 h at 37 °C as described previously [32], and seeded with 10^9^-fold diluted brain material of the animal from the second passage of synthetic strain S05 [11], which contained predominantly atypical PrPres. dgPMCAb was carried out for 18 rounds with 10-fold dilution between rounds. The absence of PrP^Sc^ in the final product was confirmed by failure to amplify in 6 rounds of PMCAb (Fig. 2d).

### Bioassay

Each hamster received 50 μl of inoculum intracerebrally, under 2 % O_2_/4 minimum alveolar concentration (MAC) isoflurane anesthesia. After inoculation, animals were observed daily for disease using a ‘blind’ scoring protocol. The study was carried out in strict accordance with the recommendations in the Guide for the Care and Use of Laboratory Animals of the National Institutes of Health. The animal protocol was approved by the Institutional Animal Care and Use Committee of the University of Maryland, Baltimore (Assurance Number A32000-01; Permit Number: 0215002).

### Proteinase K digestion of brain homogenates

For the PK digestion in sarcosyl, an aliquot of 10 % BH was prepared as described previously [30]. Briefly, 10 % BH was mixed with an equal volume of 4 % sarcosyl in PBS, supplemented with 50 mM Tris, pH 7.5, and digested with 20 μg/ml PK (New England BioLabs) for 30 min at 37 °C with 1000 rpm shaking using a DELFIA plate shaker (Wallac) placed in 37 °C incubator. PK digestion was stopped by adding SDS sample buffer and heating the samples for 10 min in a boiling water bath. Samples were loaded onto NuPAGE 12 % Bis-Tris gels, transferred to PVDF membrane, and probed with 3F4 or SAF-84 antibodies.

### Analysis of conformational stability and Proteinase K resistance

10 % brain homogenate was diluted 10 times into PMCAb conversion buffer, then supplemented with an equal volume of GdnHCl solution in PBS to a final concentration of GdnHCl ranging from 0.4 to 4 M, and incubated at room temperature for 1 h. Next, nine volumes of 2 % sarkosyl in PBS were added to all samples followed by 1 h incubation at room temperature, and then the samples were treated with 20 μg/mL PK for 1 h at 37 °C with shaking. The digestion was stopped with 2 mM PMSF, and the proteins were precipitated in four volumes of ice-cold acetone, incubated overnight at −20 °C, and subsequently centrifuged for 30 min at 16000 x g. Pellets were dried for 30 min, resuspended in 1 × SDS-sample buffer, loaded into NuPAGE 12 % bisTris gels, then transferred to PVDF membrane, and stained with 3F4 or SAF-84 antibody. To analyze PK resistance, 10 % BH was diluted 10-fold into PMCAb conversion buffer, then supplemented with 0.2 % SDS and digested with serially diluted glycerol-free PK (Sigma, Cat. # P6556) for 1 h at 37 °C. The digestion was terminated by addition of SDS-sample buffer and heating the samples for 10 min in a boiling water bath.

### PNGase F treatment of PrP^Sc^

10 % brain homogenate was diluted with an equal volume of 4 % sarcosyl in PBS, pH 7.4, digested with 20 μg/ml PK as described above, deglycosylated following the procedure described previously [10], and assayed by Western blot with 3 F4 antibody.

### 2D electrophoresis

25 μL samples were heated for 10 min in a boiling water bath in the presence of gel loading buffer, solubilized for 1 h at room temperature with 200 μL solubilization buffer (8 M Urea, 2 % (wt/vol) CHAPS, 5 mM TBP, 20 mM TrisHCl pH 8.0), then alkylated by adding 7 μL of 0.5 M iodoacetamide and incubated for 1 h at room temperature in the dark. Then, 1150 μL of ice-cold methanol was added and samples were incubated for 2 h at −20 °C. After centrifugation at 16,000 g at 4 °C, supernatant was discarded and the pellet was re-solubilized in 160 μL rehydration buffer (7 M urea, 2 M thiourea, 1 % (wt/vol) DTT, 1 % (wt/vol) CHAPS, 1 % (wt/vol) Triton X-100, 1 % (vol/vol) ampholyte, trace amount of Bromophenol Blue). Fixed immobilized pre-cast IPG strips (cat. # ZM0011, Life Technologies, Carlsbad, CA) with a linear pH gradient 3–10 were rehydrated in 155 μL of resulting mixture overnight at room temperature inside IPG Runner cassettes (cat. # ZM0008, Life Technologies). Isoelectrofocusing (first dimension separation) was performed at room temperature with rising voltage (175 V for 15 min, then 175–2,000 V linear gradient for 45 min, then 2,000 V for 30 min) on Life Technologies Zoom Dual Power Supply using the XCell SureLock Mini-Cell Electrophoresis System (cat. # EI0001, Life Technologies). The IPG strips were then equilibrated for 15 min consecutively in (i) 6 M Urea, 20 % (vol/vol) glycerol, 2 % SDS, 375 mM Tris-HCl pH 8.8, 130 mM DTT, and (ii) 6 M Urea, 20 % (vol/vol) glycerol, 2 % SDS, 375 mM Tris-HCl pH 8.8, 135 mM iodoacetamide, and loaded on 4–12 % Bis-Tris ZOOM SDS-PAGE pre-cast gels (cat. # NP0330BOX, Life Technologies). For the second dimension, SDS-PAGE was performed for 1 h at 170 V. Immunoblotting was performed as described elsewhere, blots were stained using 3F4 or SAF-84 antibody.

### Histopathological study

The brains of two animals from the second passage of F^0.5^-derived atypical PrPres were evaluated for the presence of spongiform changes and intensity of PrP immunostaining. Formalin-fixed brain halves divided at the midline (left hemisphere) were processed for hematoxylin-eosin (H&E) stain and immunohistochemistry staining using the mouse monoclonal anti-PrP antibody 3F4 (1:1000, Covance). Blocks were treated in formic acid (96 %) before being embedded in paraffin. For detection of disease-associated PrP, blocks were pretreated by 15 min hydrated autoclaving at 121 °C in trisodium citrate buffer, pH 6.0 with 0.05 % Tween 20, followed by 5 min in 88 % formic acid.

## Results

### Triggering atypical PrPres i*n vitro* is a generic property of rPrP fibrils

In previous studies, upon inoculations into animals, rPrP fibrils were shown to induce misfolding of PrP^C^ into atypical PrPres [11, 12]. To test whether this process could be recapitulated *in vitro*, rPrP fibrils were prepared according to the protocols used in previous studies for making fibrils that induced transmissible prion diseases [10–12, 29]. Regardless of the experimental protocol, all fibril preparations triggered atypical PrPres in a PMCAb format that used partially deglycosylated PrP^C^ substrate, referred to as dgPMCAb (Fig. 2a) [26]. Notably, dgPMCAb was previously found to favor selective replication of brain-derived atypical PrPres over PrP^Sc^ [26]. When serial PMCAb reactions, instead of dgPMCAb, were seeded with the same fibril preparations, no PK-resistant products were found (Fig. 2a). This result is in agreement with the previous studies that rPrP fibrils do not seed PrP^Sc^ nor contain small amounts of PrP^Sc^ amplifiable by PMCAb [11, 12, 33]. rPrP fibril-induced formation of atypical PrPres in dgPMCAb was robust and highly reproducible (Fig. 1b). No PK-resistant products were found in non-seeded dgPMCAb control reactions (Fig. 1b). For further experiments, we choose rPrP fibrils prepared in 0.5 M GdnHCl (abbreviated as F^0.5^), as they were the most effective in inducing prion disease in animals [11].

### *In vitro* preparation of atypical PrPres materials for animal bioassay

To prepare atypical PrPres for inoculation, dgPMCAb reactions were seeded with F^0.5^ fibrils and subjected to ten serial rounds. The products of the 10^th^ rounds, abbreviated as F^0.5^-induced atypical PrPres, were inoculated into Syrian hamsters. In parallel to the F^0.5^-induced atypical PrPres, brain-derived atypical PrPres material free of PrP^Sc^ was also produced for inoculation using the following strategy. When subjected to a serial dgPMCAb, atypical PrPres readily amplifies, whereas PrP^Sc^ does not. To ensure the absence of PrP^Sc^ the following procedures were followed. First, S05 brain material of an asymptomatic animal containing predominantly atypical PrPres was used to seed dgPMCAb [11]. Second, for seeding, this material was diluted 10^9^-fold that corresponds to the limiting dilution of atypical PrPres (Fig. 1c) (dgPMCAb titration of atypical PrPres material is in Additional file 1: Figure S1). Third, the substrate for dgPMCAb was treated with RNase, a procedure that selectively amplifies atypical PrPres but not PrP^Sc^ [26]. Fourth, 18 serial dgPMCAb rounds with 10-fold dilutions between rounds were conducted to completely dilute out any initial PrP^Sc^ material, if such would still present after the initial 10^9^-fold dilution (Fig. 2c). Moreover, to confirm absence of PrP^Sc^ in the preparation of brain-derived atypical PrPres amplified in dgPMCAb, the products of the 18^th^ dgPMCAb round were subjected to six serial PMCAb rounds (Fig. 2d). Previously, we showed that six PMCAb rounds were sufficient for amplifying a single PrP^Sc^ particle to the amounts detectible by Western blot [34]. As expected, no PrP^Sc^ was detected by Western blot after six PMCAb rounds in the preparation of atypical PrPres (Fig. 2d).

### F^0.5^-induced atypical PrPres propagates in vivo and gives rise to PrP^Sc^

**Fig. 3:**
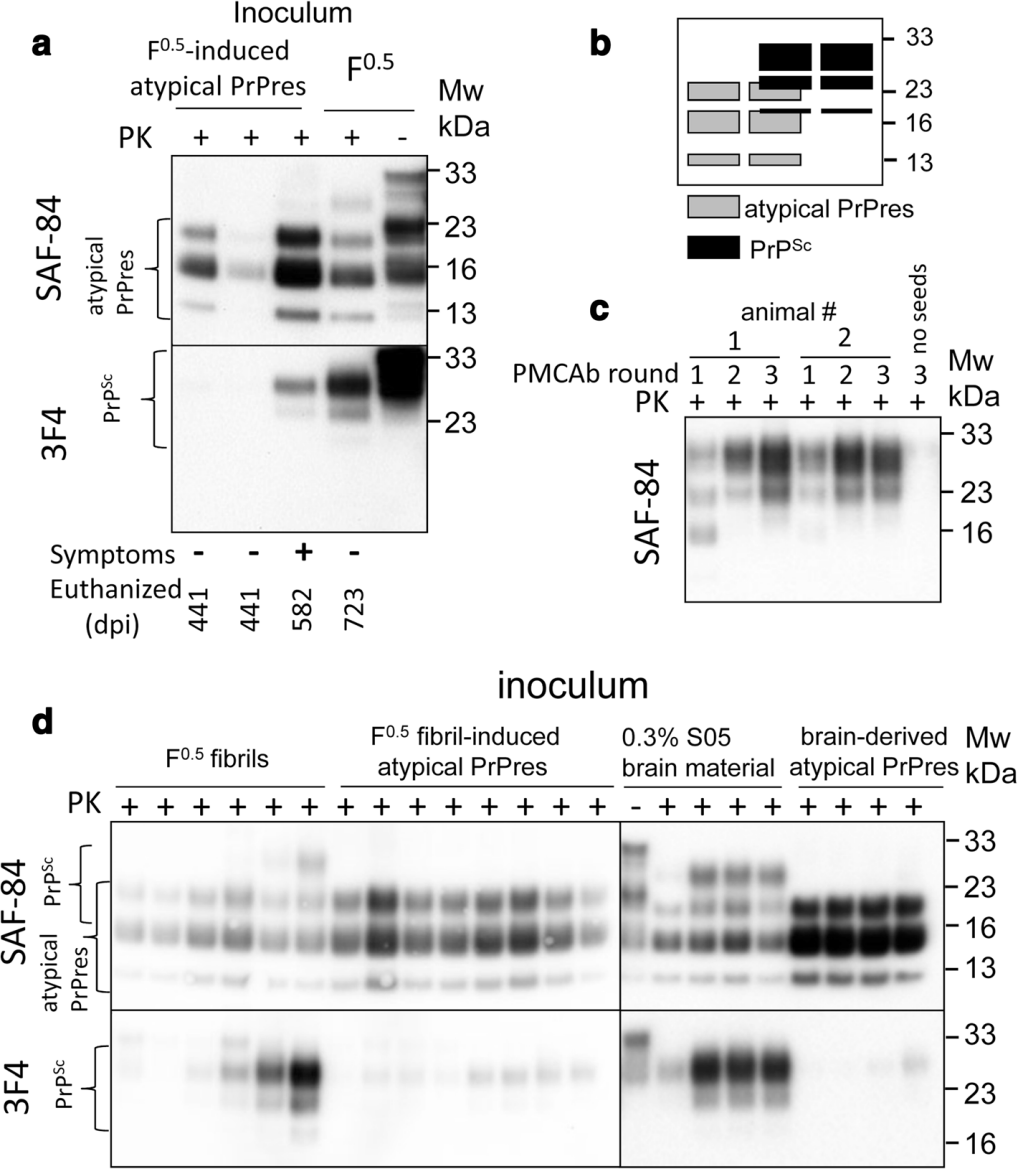
Analysis of brain materials from the first passage. **a** Western blots of brain materials from animals inoculated with F^0.5^-induced atypical PrPres and sacrificed at 441 days post-inoculation. Brain material from animal inoculated directly with F^0.5^ is included as a reference. **b** Schematic representation of the PK resistance profile showing overlap between atypical PrPres and PrP^Sc^, where atypical PrPres and PrP^Sc^ are represented by gray and black boxes, respectively. **c** Western blot analysis of PK-resistant materials amplified in serial PMCAb reactions seeded with brain materials from animals inoculated with F^0.5^-induced atypical PrPres and sacrificed at 441 days. **d** Western blots of brain materials from animals inoculated with F^0.5^ fibrils, F^0.5^ fibril-induced atypical PrPres, 0.3 % S05 brain material or brain-derived atypical PrPres prepared as described in Fig. 2c. Western blots were stained with SAF-84 or 3F4 antibody as indicated

F^0.5^-induced atypical PrPres as well as S05 brain-derived atypical PrPres amplified in dgPMCAb were inoculated into two groups of wild type animals to test whether atypical PrPres is able to give rise to PrP^Sc^ (Table 1, Fig. 2). The results of these inoculations were compared to the direct inoculation of F^0.5^ fibrils (Table 1). In addition, S05 brain material, used to seed dgPMCAb for production of brain-derived atypical PrPres, was also inoculated after dilution to 0.3 % to match the amount of atypical PrPres, after 18 rounds of dgPMCAb (Table 1). Two out of ten animals inoculated with F^0.5^-induced atypical PrPres were sacrificed at 441 days post inoculation. Atypical PrPres was found in their brains, proving that fibril-derived atypical PrPres generated in vitro is able to propagate in vivo (Fig. 3a). Because PK-resistant bands of atypical PrPres and PrP^Sc^ overlap (Fig. 3b), discrimination between the two forms required staining with 3F4 (detects only PrP^Sc^) and SAF-84 (stains both PrP^Sc^ and atypical PrPres). While no PrP^Sc^ was found by Western blot at 441 days post inoculation (Fig. 3a), serial PMCAb revealed that small amounts of PrP^Sc^ were present in brains of these animals (Fig. 3c). Later on, at 476 days postinoculation, one out of eight animals remaining in this group developed clinical signs similar to those previously observed for synthetic hamster strains. The disease progressed very slowly just like the slow progression in hamsters inoculated with other synthetic strains [11, 12, 30]. This animal was euthanized 582 days postinoculation. In addition to atypical PrPres, PrP^Sc^ was detected by Western blot in the brain of the symptomatic animal (Fig. 3a). The remaining, asymptomatic animals were euthanized between 606 and 636 days postinoculation (Table 1). None of the animals from the other groups showed clinical disease, albeit atypical PrPres and PrP^Sc^ were found in animals of all groups. In summary, animal bioassay proved that atypical PrPres generated in vitro by seeding dgPMCAb with rPrP fibrils replicated in vivo and gave rise to PrP^Sc^.

**Table 1:** Bioassay of rPrP amyloid fibrils

Inoculum	n_s_/n_t_^a^	n_PKres_/n_t_^b^	Euthanized, days post inocul.
F^0.5^ rPrP fibrils (1st passage)	0/7	6/7	723
F^0.5^ fibril-induced atypical PrPres (1st passage)	1/10	10/10	2 at 441, 582, 2 at 630, 5 at 636
Brain-derived atypical PrPres amplified in dgPMCA	0/4	4/4	606
atypical PrPres from S05 brain^c^	0/4	4/4	606
BH from animal inoculated with F^0.5^ fibril-induced atypical PrPres (2nd passage)	4/6	6/6	630

^a^ number of animals with clinical signs over the total number of animals survived to the end of the experiment

^b^ number of animals with PK-resistant PrP in BHs detectible by Western blot over the total number of animals survived to the end of the experiment

^c^ 0.3 % brain material from the second passage of SO5 [11]

### Transition from atypical PrPres to PrP^Sc^ and a competition between atypical PrPres and PrP^Sc^

To elucidate relationship between atypical PrPres and PrP^Sc^, the relative amounts of two states were compared by Western blot in four groups: animals inoculated with F^0.5^ fibrils, F^0.5^-induced atypical PrPres, S05 brain-derived atypical PrPres amplified in dgPMCAb, and with diluted S05 brain material not subjected to dgPMCA (Fig. 3b, d). Several observations could be made. First, atypical PrPres were found in all animals from all four groups, whereas PrP^Sc^ were observed only in a fraction of animals and in smaller amounts than atypical PrPres (Fig. 3d). Such dynamics support the hypothesis that atypical PrPres is the first product of PrP^C^ misfolding, that it faithfully propagates in vivo independently of PrP^Sc^ and that it precedes PrP^Sc^. Second, small amounts of PrP^Sc^ detectible by Western blot were found in the brains of animals infected with the preparations of brain-derived atypical PrPres amplified in dgPMCAb that lacked PrP^Sc^. This result suggests that atypical PrPres not only precedes, but also gives rise to PrP^Sc^. Not surprisingly, the highest amounts of PrP^Sc^ were found in the group inoculated with S05 brain material, since in addition to atypical PrPres it contained small amount of PrP^Sc^. Brain-derived atypical PrPres enriched through dgPMCA lacked PrP^Sc^ and thus resulted in formation of very low amounts of PrP^Sc^ upon inoculation, similar to the amount of PrP^Sc^ found in the brains inoculated with F^0.5^-induced atypical PrPres. Interestingly, without the interference of PrP^Sc^, atypical PrPres accumulated to a greater degree. Unexpectedly, PrP^Sc^ amounts accumulated in the brains of animals inoculated with F^0.5^ fibril-induced or brain-derived atypical PrPres did not exceed the amount of PrP^Sc^ found in some animals inoculated with F^0.5^ fibrils. This observation, however, is consistent with the two-step mechanism, according to which (i) atypical PrPres gives rise to PrP^Sc^ in a stochastic manner and (ii) PrP^Sc^ competes with atypical PrPres (Fig. 1). As judged from PK-resistance and conformational stability assays, both PrP^Sc^ and atypical PrPres were extremely resistant to PK degradation and GdnHCl-induced denaturation (Additional file 2: Figure S2). Such high conformational stability and stability to proteolytic degradation explains why both states can co-exist and compete with each other for a long time. Upon inoculation of high amounts of preformed atypical PrPres, PrP^Sc^ arises, but has a low chance of outcompeting atypical PrPres in the 1^st^ passage.

### Atypical PrPres and PrP^Sc^ are structurally different

**Fig. 4:**
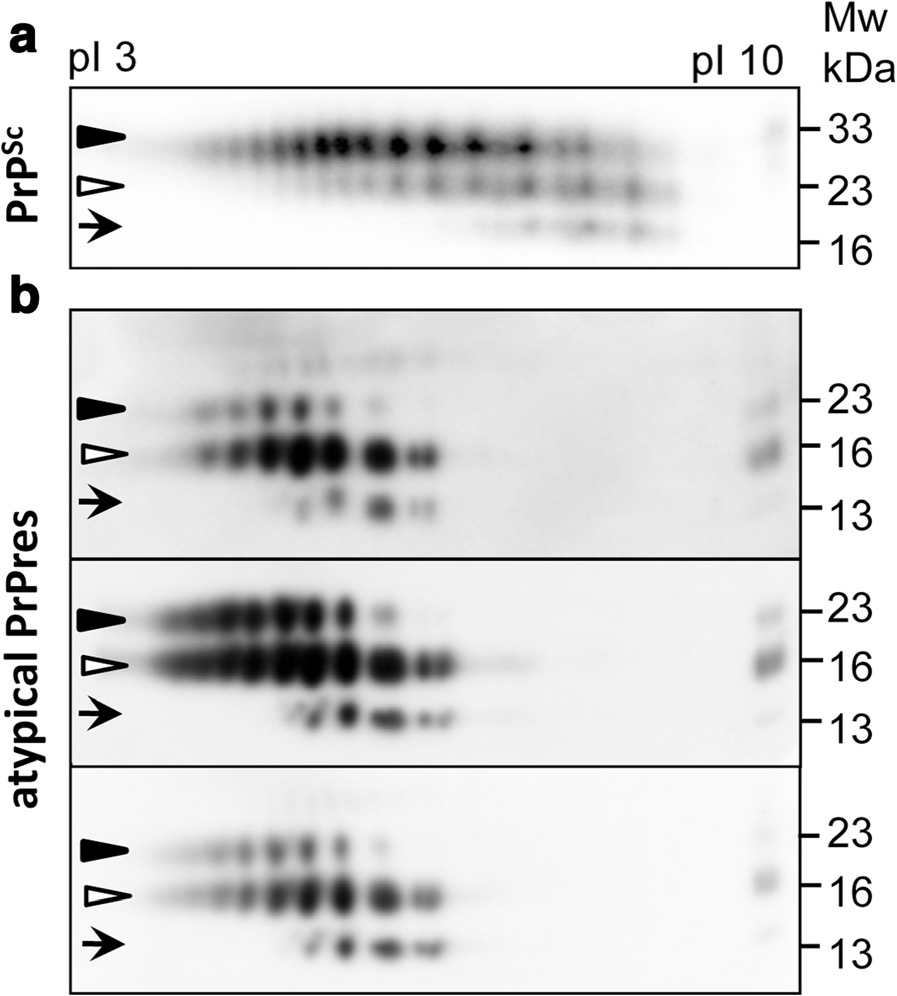
2D analysis of sialylation status of PrP^Sc^ and atypical PrPres. 2D analysis of charge distribution of S05 brain-derived PrP^Sc^ **(a)** and atypical PrPres from animals inoculated with F^0.5^ fibrils (**b**, upper panel), atypical PrPres induced in animals by inoculating brain-derived atypical PrPres amplified in dgPMCAb (**b**, middle panel), or asymptomatic S05 animals that lacked PrP^Sc^ (**b**, lower panel). Black triangles, white triangles and arrows mark di-, mono-, and unglycosylated glycoforms, respectively. The distributions of all three glycoforms of atypical PrPres are shifted toward acidic pH relative to those of PrP^Sc^ due to differences in number of charged amino acid residues and, accordingly, pIs between atypical PrPres and PrP^Sc^. All samples were treated with PK. Western blots were stained with 3F4 **(a)** and SAF-84 **(b)**

Previous work established that atypical PrPres and PrP^Sc^ have different structures, based on different the length of the PK-resistant core, glycoform ratios and different RNA-dependency for amplification [11, 12, 26, 27]. The predominantly monoglycosylated composition of atypical PrPres suggests that the diglycosylated glycoforms are excluded from atypical PrPres, presumably due to strong structural constraints and electrostatic repulsion between negatively charged terminal sialic acid residues on N-linked glycans. Assuming that structural constraints are much stronger in atypical PrPres than PrP^Sc^, we propose that diglycosylated, hypersialylated molecules should be selectively excluded in atypical PrPres. To test this hypothesis, distribution of charge isoforms that reflects sialylation patterns of individual PrP molecules within PrP^Sc^ or atypical PrPres were analyzed using 2D (Fig. 4) [35]. In both states, unglycosylated molecules showed more than one charge isoforms (Fig. 4). This charge heterogeneity is largely attributed to the structural heterogeneity of the GPI anchors [36]. As expected, in PrP^Sc^ the distribution of monoglycosylated isoforms extended into acidic pH far beyond that of unglycosylated isoforms, and the distribution of diglycosylated isoforms extended into acidic pH beyond monoglycosylated isoforms (Fig 4a). This is because diglycosylated isoforms can incorporate more sialic acid residues per molecule than monoglycosylated isoforms. In atypical PrPres, the distribution of monoglycosylated isoforms also extended into acidic pH far beyond that of unglycosylated isoforms (Fig. 4b). However, diglycosylated isoforms did not extend beyond monoglycosylated isoforms, illustrating that atypical PrPres excluded diglycosylated molecules with high sialylation levels. Atypical PrPres of three different origins were compared by 2D: one induced in animals by inoculating F^0.5^ fibrils, atypical PrPres induced by inoculating brain-derived atypical PrPres amplified in dgPMCAb, and atypical PrPres from asymptomatic S05 animals [11]. They all showed very similar if not identical sialylation patterns characterized by the absence of hypersialylated diglycosylated molecules, a pattern that was notably different from that of PrP^Sc^ (Fig. 4).

### Two alternative biochemical pathways produce synthetic strains with similar disease phenotype

Next, we tested whether *in vitro*-generated atypical PrPres results in a disease phenotype similar to those previously described for the synthetic strains triggered by inoculation of rPrP fibrils directly. Brain material from the animal inoculated with F^0.5^-induced atypical PrPres and euthanized at 441 days post inoculation was used for the second passage. Using animal euthanized at the early time point allowed initiating the second passage approximately 200 days before the remaining animals from this group were euthanized. In the second passage, clinical disease was observed in 4 out of 6 animals after 500 dpi (Table 1). In agreement with the slow disease progression of synthetic hamster strains described previously [11, 12], the clinical disease here progressed very slowly. Animals approached terminal stages approximately 120 days after the first clinical signs and were euthanized at 630 days due to the old age. Animals displayed the same set of clinical symptoms including rough coat, dry skin and obesity as was previously observed for the synthetic strains [10–12].

**Fig. 5:**
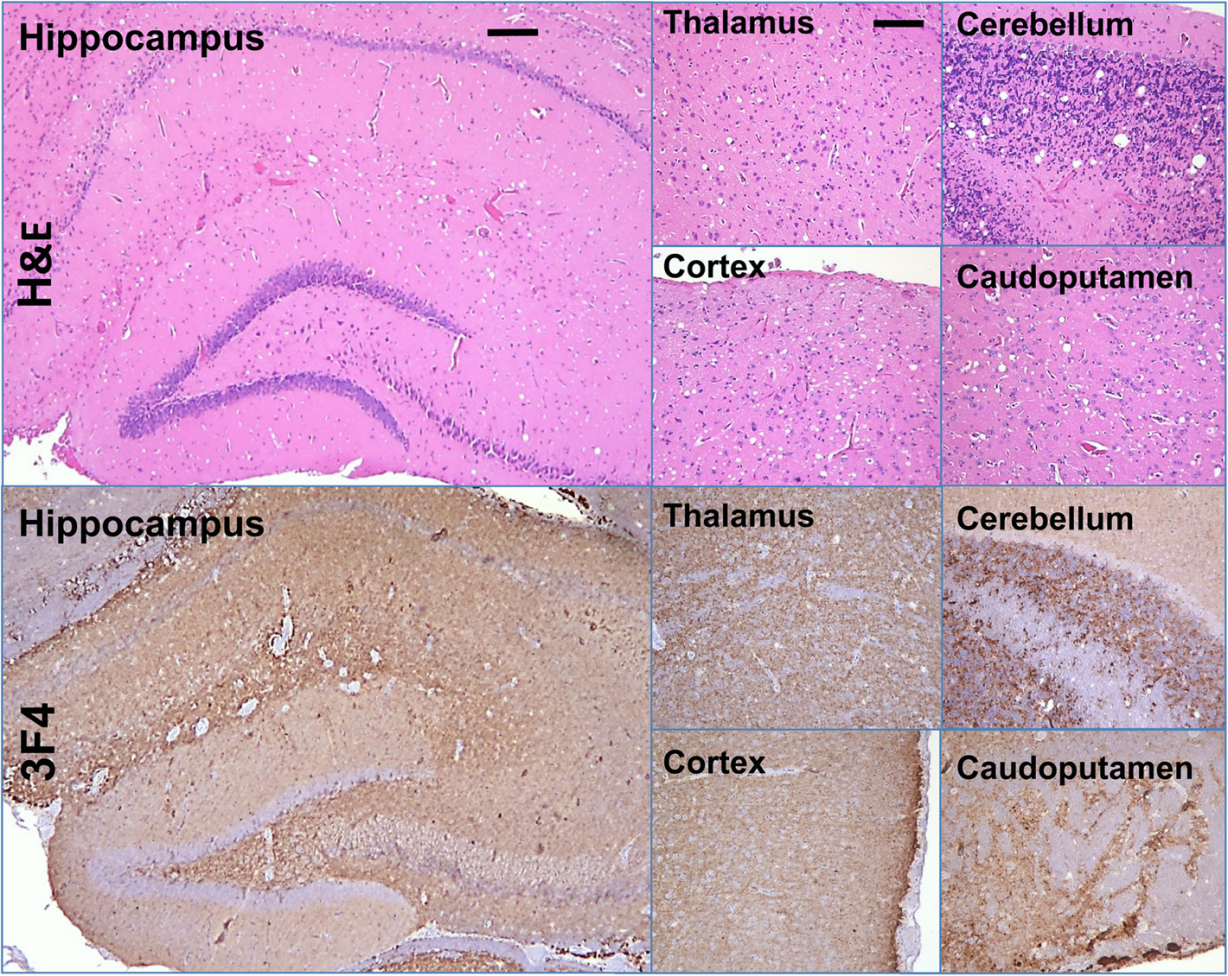
Histopathological analysis. Spongioform changes stained with hematoxylin and eosine (upper panels) or PrP deposisiton stained with 3F4 antibody (lower panels) in hippocampus, thalamus, cerebellum, frontal cortex or caudoputamen in animals from the second passage of F^0.5^-induced atypical PrPres that developed clinical disease. Scale bars = 200 μm

**Fig. 6:**
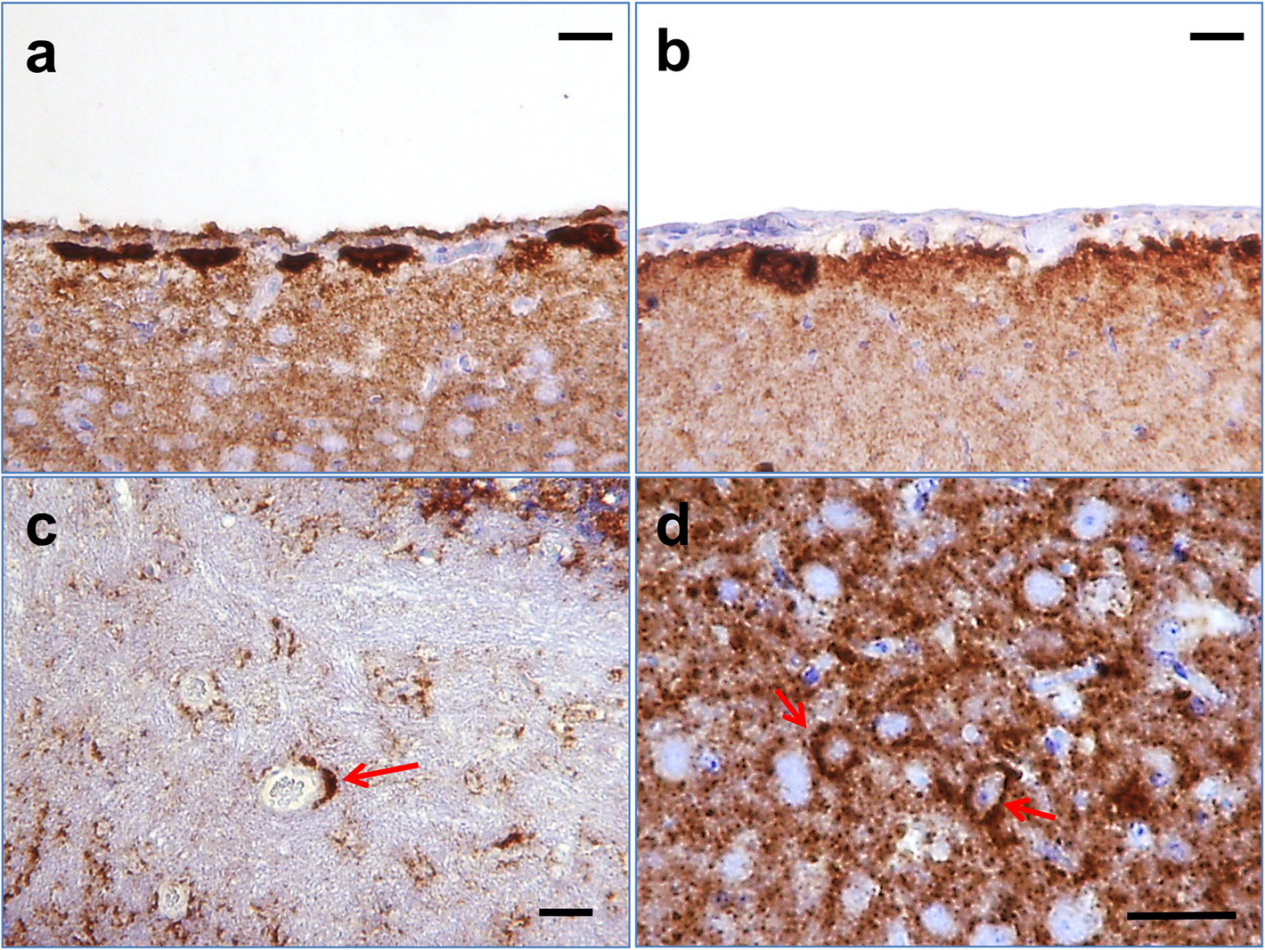
Plaques, perineuronal, perivascular or diffuse PrP depositions. PrP immunoreactivity stained with 3F4 antibody in subependymal (**a**), subpial (**b**), cerebellum white matter (**c**), or deeper layer of cortex (**d**) in animals from the second passage of F^0.5^-induced atypical PrPres that developed clinical disease. Red arrows point at perivascular deposition in **c** or perineuronal deposition in **d**. Scale bars = 50 μm

Consistent with TSE, histopathological analysis of animals from the second passage revealed a high degree of spongiform vacuolation in multiple brain areas including cerebellum, cortex, caudoputamen and thalamus (Fig. 5). Diffuse, synaptic PrP immunoreactivity was observed in the aforementioned subregions (Fig. 5). Large PrP^Sc^ plaques were observed in the periventricular subependymal region (Fig. 6), which is a hallmark of hamster synthetic strains including S05 [11]. Perineuronal PrP deposits were found in the thalamus, basal ganglia, and deeper layers of the cortex (Fig. 6). Overall, the type and brain regions of PrP depositions were consistent with depositions observed in the second passage of S05 strain produced upon direct inoculation of F^0.5^ fibrils [11].

**Fig. 7:**
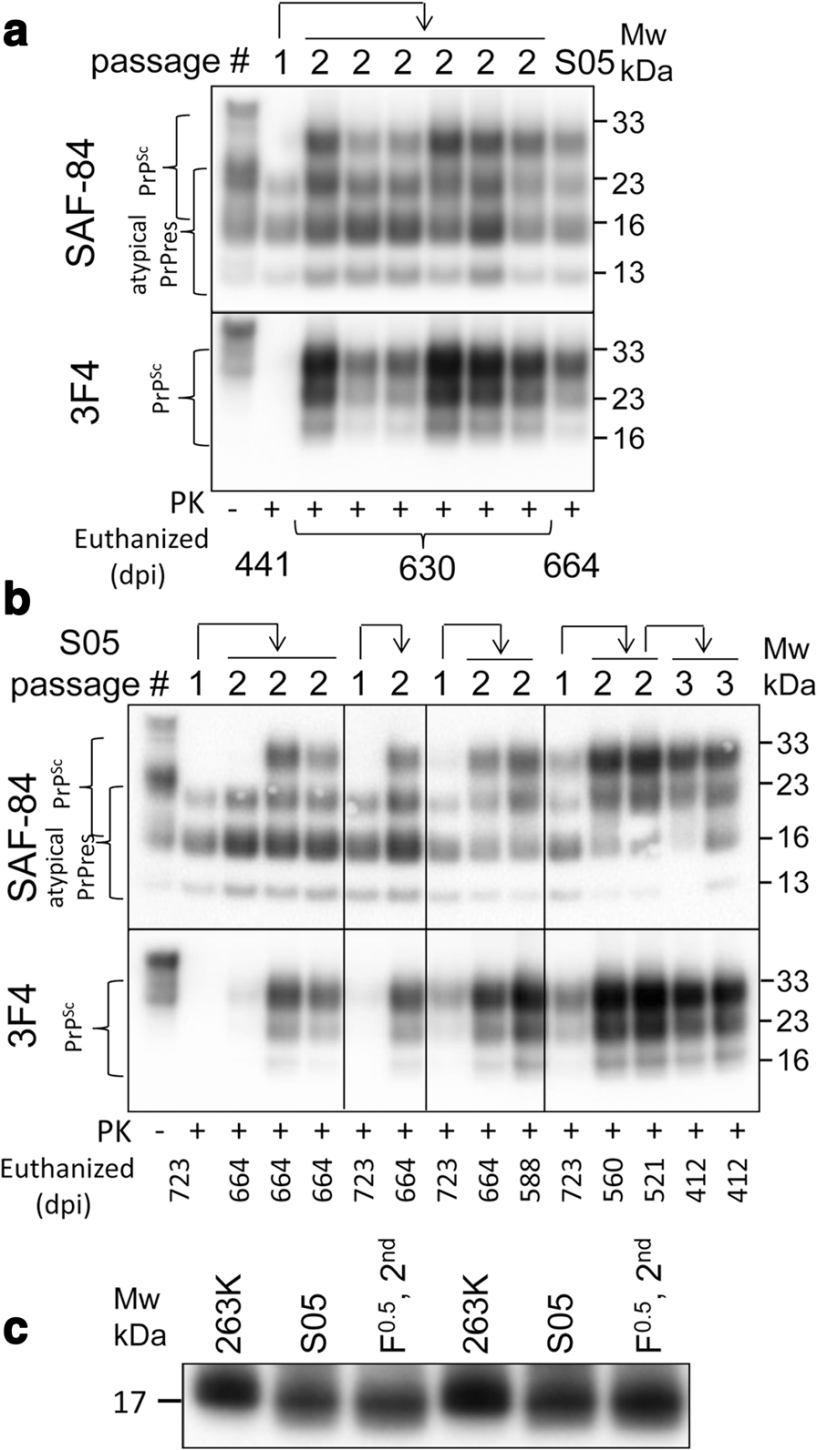
Dynamics between atypical PrPres and PrP^Sc^ in animals from the first and second passages. **a** Western blots of brain materials from animals of the first and second passages of F^0.5^-induced atypical PrPres. Brain material from an animal of the second passage of F^0.5^ (S05) included as a reference. **b** Western blots of brain materials from animals of the first, second and third passages of F^0.5^ fibrils. Arrows indicate animals that were used for preparing inoculums for the subsequent passages. Western blots were stained with SAF-84 or 3F4 antibody as indicated. **c** Brain materials from 263K- or S05-inoculated animals or second passage of F^0.5^ fibrils were treated with PNGase and PK, analyzed by Western blot and stained with 3F4

Analysis of brain material from the 2^nd^ passage of F^0.5^-induced atypical PrPres revealed increased accumulation of PrP^Sc^, in addition to propagation of atypical PrPres (Fig. 7a). The dynamics between atypical PrPres and PrP^Sc^ were very similar to those observed during serial passaging of S05 strain that was produced upon inoculation of F^0.5^ fibrils directly. In the second passage of F^0.5^ the amounts of PrP^Sc^ increased while atypical PrPres was still able to replicate (Fig. 7b). For S05, the amounts of PrP^Sc^ formed in the course of the second passage were variable and depended on the ratio of PrP^Sc^ to atypical in the inoculums (Fig. 7b). The dynamics between the two forms is consistent with the hypothesis that the two forms compete with each other and that PrP^Sc^ replaces atypical PrPres over time.

PNGase treatment revealed that the sizes of the PK-resistant cores were the same for PrP^Sc^ from the second passages of F^0.5^-induced atypical PrPres and the second passage of S05, both of which were ~0.5-1 kDa shorter than that of the 263K strain (Fig. 7c). In summary, the disease phenotype observed in a second passage of F^0.5^-induced atypical PrPres was very similar, if not identical to, that described in previous studies for S05 [11].

## Discussion

The results in the current study are important for the following reasons. First, this work illustrates that transmissible prion disease with very similar if not identical disease phenotypes can be generated *via* two experimental procedures that utilize alternative biochemical pathways. The initial step of both pathways involves an *in vitro* conversion of rPrP into an amyloid fibrilar state. However, the first procedure involves direct inoculation of rPrP fibrils into animals, whereas the second protocol relies on production of atypical PrPres *in vitro* in reactions seeded with rPrP fibrils. Second, this study provides the first direct illustration that preparations of atypical PrPres free of PrP^Sc^ produced transmissible diseases when inoculated into wild type animals. Atypical PrPres is a self-replicating, transmissible, but clinically silent state. Third, the current work provides strong support for the mechanism established in previous studies and presented in Fig. 1 that atypical PrPres is an alternative transmissible state, which is also a precursor of PrP^Sc^ [11, 12]. Fourth, the current study demonstrates that atypical PrPres generated *in vitro* using dgPMCAb is an adequate model for atypical PrPres formed in brain. Indeed, inoculation of *in vitro-*generated F^0.5^-induced atypical PrPres led to the *de novo* formation of atypical PrPres in animals. Moreover, both F^0.5^-induced atypical PrPres and brain-derived atypical PrPres gave rise to PrP^Sc^ upon serial transmission. In both atypical PrPres states, F^0.5^-induced and brain-derived, monoglycosylated PrP was the predominant glycoform and hypersialylated diglycosylated isoforms were missing. This is in contrast to the glycosylation pattern of all currently known hamster strains of synthetic or natural origin, in which diglycosylated PrP is the predominant glycoform [37]. In support of previous studies, this work argues that atypical PrPres can be faithfully and selectively propagated in the dgPMCAb [26].

The mechanism in Fig. 1 postulates that evolution of synthetic prions involves two main steps. Completing the first step, which involved a transition from rPrP fibrils to atypical PrPres, *in vitro* instead of in vivo did not accelerate the disease development. This result suggests that the major barrier on a pathway toward PrP^Sc^ is associated with the second step, i.e. generation of PrP^Sc^ via deformed templating events, which are believed to be rare and stochastic. The data on the dynamics between the two forms suggest that atypical PrPres and PrP^Sc^ compete with each other (Figs. 3 and 7). Atypical PrPres gives rise to PrP^Sc^ in stochastic events that might occur earlier or later in the course of infection. In experiments where the first PrP^Sc^ arises from atypical PrPres before a critical amount of atypical PrPres is accumulated, PrP^Sc^ is able to propagate efficiently and rapidly replaces atypical PrPres (Fig. 3d). If PrP^Sc^ appears later in the course of the infection when the amount of atypical is above a certain threshold, PrP^Sc^ replication is slowed down. Inoculation of atypical PrPres material, which was selectively amplified in dgPMCA and free of PrP^Sc^, proved its ability to produce PrP^Sc^ in animals. However, it also provided a selective advantage to the propagation of atypical PrPres conformations. Under such inoculation conditions, PrP^Sc^ accumulation during the first passage was very weak. Several passages might be needed for the PrP^Sc^ to rich a level, at which it exceed and effectively outcompete atypical PrPres as it was observed with S05 [11]. With the atypical PrPres inhibiting propagation of the disease-related forms we have an interesting case of a “protective misfolding”, where PrP^C^ recruitment into an alternative misfolded, PK-resistant, non-toxic, self-replicating state delays development of a deadly disease.

Atypical PrPres is remarkable with respect to its biochemical and biological properties. While fully transmissible in animal studies, this is a clinically silent state, which in the absence of PrP^Sc^ does not cause prion diseases or clinical symptoms [11, 12, 25]. Atypical PrPres forms large plaques and diffuse synaptic oligomers, however, neither is associated with neurotoxic or pathogenic effects [25]. Atypical PrPres is structurally different from PrP^Sc^, as evident from the differences in length of PK-resistant fragments, glycoform ratios and RNA-dependency of their amplification *in vitro* [11, 12, 26, 27]*.* Moreover, the current study established that atypical PrPres and PrP^Sc^ exhibit significantly different sialylation patterns. In accordance with previous work [35], PrP^Sc^ was found to contain PrP molecules with a wide range of sialylation levels encompassing molecules with both highly and moderately sialylated glycans. In contrast to PrP^Sc^, atypical PrPres does not recruit PrP^C^ with high levels of sialylation. Negatively charged sialic acid residues create electrostatic repulsion between neighboring N-linked glycans. Differences in sialylation status suggest that atypical PrPres has much stronger structural constraints for recruiting diglycosylated or heavily sialylated molecules than PrP^Sc^. As a result, only a fraction of PrP^C^ that is acceptable as a substrate for PrP^Sc^ is also acceptable to atypical PrPres. Differences in sialylation offer one of the possible explanations why atypical PrPres replicates slower than PrP^Sc^ and why PrP^Sc^ can eventually outcompete atypical PrPres. Differences in sialylation pattern could also be responsible for the lack of toxicity by atypical PrPres. This hypothesis remains to be tested. Alternatively, the lack of neurotoxicity could be due to the absence of structural determinants associated with toxic effects. Another hypothesis that would be interesting to consider in future studies is whether sialylation deficiency observed in certain genetic or metabolic disorders can facilitate conversion of PrP^C^ into alternative self-replicating states similar to atypical PrPres or CTF12/13 associated with sCJD.

A number of atypical or short PK-resistant fragments has been identified in human and animal prion diseases. PK-resistant fragments referred to as CTF12/13 that encompass residues 154/156-231 and 162/167-231 were found in the majority of patients with sCJD [38]. C-terminal PK-resistant species of similar size were also found in iatrogenic CJD [39] and in mice infected with mouse-passaged hamster scrapie [40]. Furthermore, similar C-terminal PK-resistant fragments were found in atypical bovine spongiform encephalopathy, which is believed to be sporadic in origin [41], and in certain types of ovine scrapie [42]. While the size and position of PK-resistant fragments of atypical PrPres described in the current study are very similar to those found in human and animal diseases of natural origin, it is not clear whether they are structurally related or play similar role in disease etiology. Moreover, the relationship of the C-terminal fragments found in sCJD, iatrogenic CJD or in atypical bovine spongiform encephalopathy to PrP^Sc^ is also uncertain. Taking into account results presented here, one can speculate that CTF12/13 species are precursors of PrP^Sc^ and important for the etiology of sporadic CJD. Testing this hypothesis would involve selective amplification of sCJD-derived CTF12/13 species in dgPMCAb to generate CTF12/13 material free of PrP^Sc^ and then testing whether this material induce prion diseases with sCJD phenotype in humanized mice. Comparison of the sialylation status of atypical PK resistant fragments in prion diseases of natural and synthetic origin would help to establish a relationship between them as well as their relationship with PrP^Sc^.

This work highlights the role of deformed templating in transformation and evolution of protein self-replicating states. The deformed templating model predicts emergence of new, structurally altered self-replicating states upon changes in replication environment [16]. In agreement with this prediction, treatments with anti-prion drugs or prion inhibitors were found to result in transformation of strain-specific properties and emergence of drug-resistant prions [20, 43–45].

## Conclusions

In summary, the current work illustrate for the first time that transmissible prion disease with similar disease phenotype can be produced via two alternative procedures: direct inoculation of recombinant PrP amyloid fibrils or *in vitro*-produced atypical PrPres. Moreover, this work showed that preparations of atypical PrPres free of PrP^Sc^ can give rise to transmissible diseases in wild type animals and that atypical PrPres generated *in vitro* is an adequate model for brain-derived atypical PrPres. Finally, the current study illustrates a remarkable case of competition between two transmissible states formed by the same protein, where one state being clinically silent, whereas another being neurotoxic and responsible for progression of disease. Differences in sialylation patterns between PrP^Sc^ and atypical PrPres revealed that only a fraction of PrP^C^ that is acceptable as a substrate for PrP^Sc^ is also acceptable to atypical PrPres. This explains why atypical PrPres replicates slower than PrP^Sc^ and why PrP^Sc^ can eventually outcompete atypical PrPres. Other amyloidogenic proteins that are responsible for a range of neurodegenerative disease are known to spread via prion-like mechanisms [46, 47]. It remains to be determined whether competition between clinically silent and toxic states is a general phenomenon or specific to the prion protein.

## Additional files

Additional file 1: Figure S1. Establishing a limiting dilution of atypical PrPres in S05 brain material. S05 brain material was serially diluted up to 10^13^-fold, then each dilution was used to seed serial dgPMCAb; 18 serial dgPMCAb rounds were conducted and analyzed by Western blot. Ten serial PMCAb rounds were sufficient to amplify the highest dilution of brain material that still contains atypical PrPres (10^9^-fold dilution) to the level detectible by Western blot. The reactions seeded with 10^10^-fold or higher dilutions were all negative regardless of the number of serial dgPMCAb rounds (Fig. 2c). Western blots were stained with SAF-84 antibody. (TIF 1376 kb) Additional file 2: Figure S2. PK-resistance and conformational stability of atypical PrPres and PrP^Sc^. a Analysis of PK-resistance. Brain material from S05-inoculated animals that contained predominantly PrP^Sc^ (upper panel) or atypical PrPres (lower panel) were treated with increasing concentration of glycerol-free proteinase and analyzed by Western blot. b Analysis of conformational stability. Brain materials from S05-inoculated animals that contained predominantly PrP^Sc^ (upper panel) or atypical PrPres (lower panel) were incubated with increasing concentrations of GdnHCl, digested with PK and analyzed by Western blot. Animals from the second passage of S05 were used. Western blots were stained with 3F4 or SAF-84 antibody as indicated. (TIF 1302 kb)

## Abbreviations

BSA: bovine serum albumin
sCJD: sporadic Creutzfeldt Jakob Disease
dgPMCAb: Protein misfolding cyclic amplification with partially deglycosylated substrate
dpi: days post inoculation
GdnHCl: Guanidine Hydrochloride
NBH: Normal brain homogenate
PBS: Phosphate buffered saline buffer
PK: Proteinase K
PMCAb: Protein misfolding cyclic amplification with beads
PNGase F: peptide-*N*-glycosidase F
PrP^C^: normal, cellular form of the prion protein
PrP^Sc^: disease-associate, infectious form of the prion protein
rPrP: recombinant prion protein
TSEs: Transmissible Spongiform Encephalopathies
