# Supplementary figures and images for: Two alternative pathways for generating transmissible prion disease de novo
Source: Acta Neuropathol Commun. 2015 Nov 10;3:69. doi: 10.1186/s40478-015-0248-5 (PMC4641408; doi:10.1186/s40478-015-0248-5)

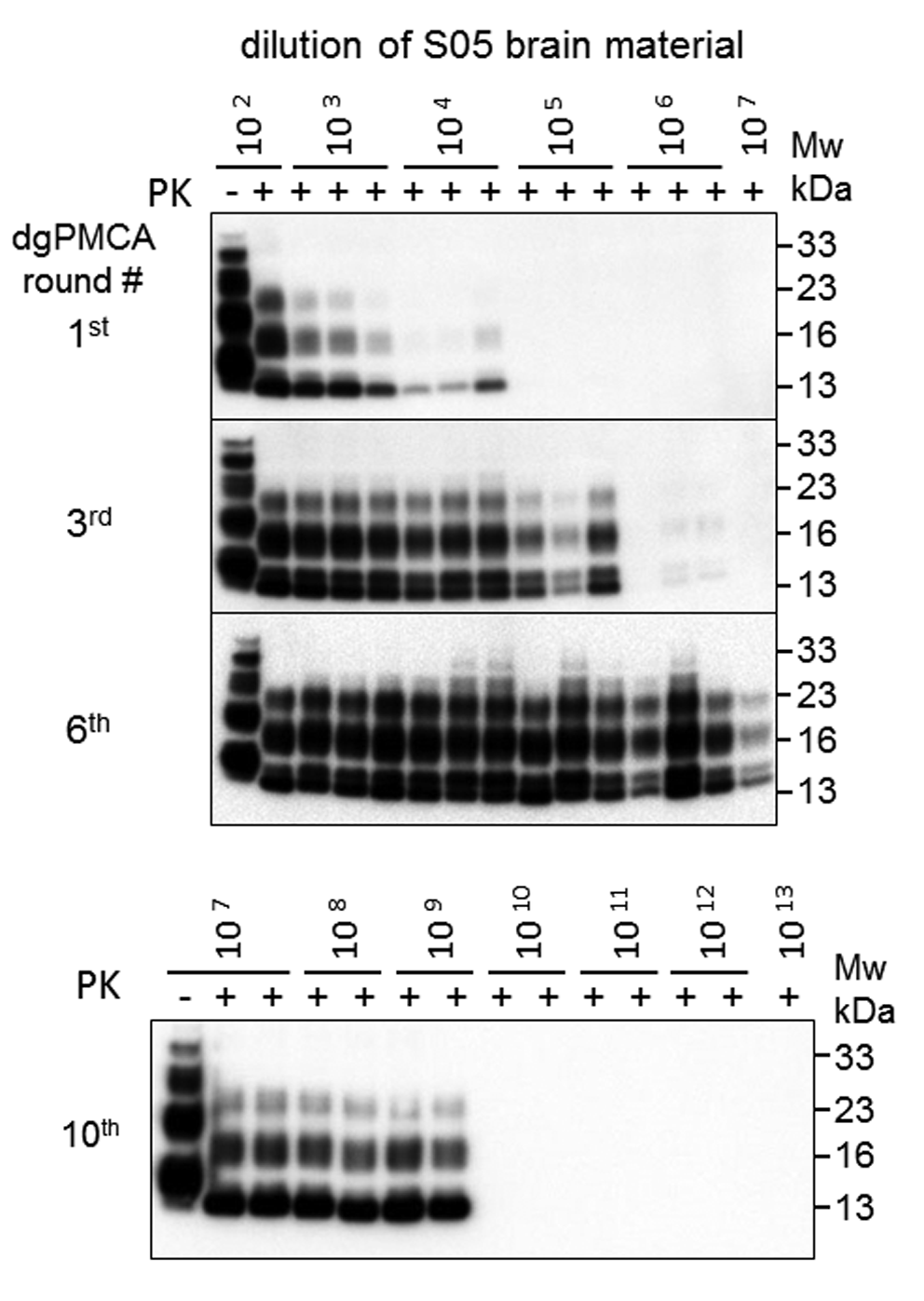

Supplement: Additional file 1: Figure S1. — Establishing a limiting dilution of atypical PrPres in S05 brain material. S05 brain material was serially diluted up to 1013-fold, then each dilution was used to seed serial dgPMCAb; 18 serial dgPMCAb rounds were conducted and analyzed by Western blot. Ten serial PMCAb rounds were sufficient to amplify the highest dilution of brain material that still contains atypical PrPres (109-fold dilution) to the level detectible by Western blot. The reactions seeded with 1010-fold or higher dilutions were all negative regardless of the number of serial dgPMCAb rounds (Fig. 2c). Western blots were stained with SAF-84 antibody. (TIF 1376 kb) [file 40478_2015_248_MOESM1_ESM.tif]

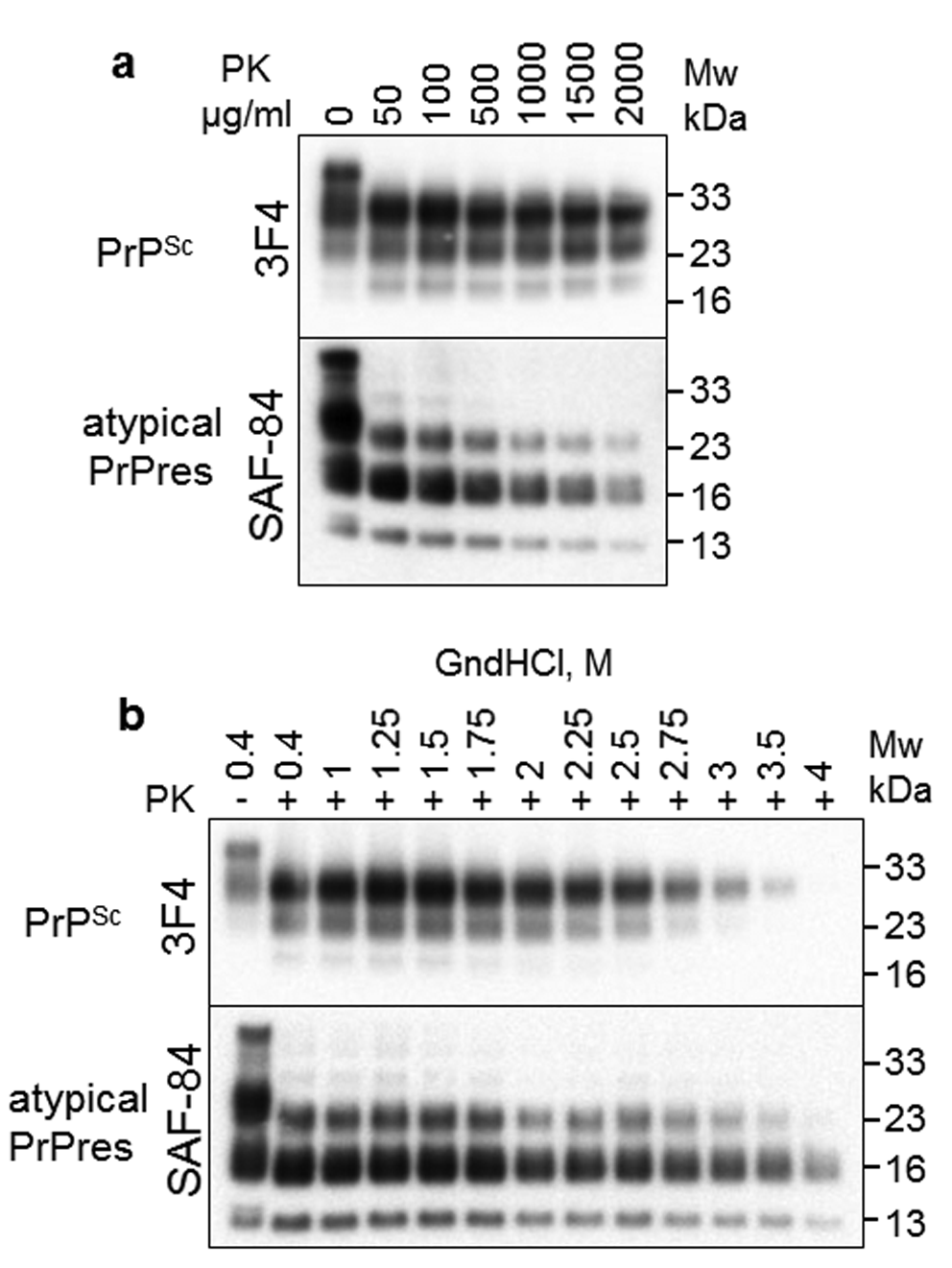

Supplement: Additional file 2: Figure S2. — PK-resistance and conformational stability of atypical PrPres and PrPSc. a Analysis of PK-resistance. Brain material from S05-inoculated animals that contained predominantly PrPSc (upper panel) or atypical PrPres (lower panel) were treated with increasing concentration of glycerol-free proteinase and analyzed by Western blot. b Analysis of conformational stability. Brain materials from S05-inoculated animals that contained predominantly PrPSc (upper panel) or atypical PrPres (lower panel) were incubated with increasing concentrations of GdnHCl, digested with PK and analyzed by Western blot. Animals from the second passage of S05 were used. Western blots were stained with 3F4 or SAF-84 antibody as indicated. (TIF 1302 kb) [file 40478_2015_248_MOESM2_ESM.tif]
